# Machine Learning Methods and Synthetic Data Generation to Predict Large Wildfires

**DOI:** 10.3390/s21113694

**Published:** 2021-05-26

**Authors:** Fernando-Juan Pérez-Porras, Paula Triviño-Tarradas, Carmen Cima-Rodríguez, Jose-Emilio Meroño-de-Larriva, Alfonso García-Ferrer, Francisco-Javier Mesas-Carrascosa

**Affiliations:** 1 Department of Graphic Engineering and Geomatics, Campus de Rabanales, University of Córdoba, 14071 Córdoba, Spain; o12pepof@uco.es (F.-J.P.-P.); ig2trtap@uco.es (P.T.-T.); ir1melaj@uco.es (J.-E.M.-d.-L.); agferrer@uco.es (A.G.-F.); 2 Centro de Investigaciones Aplicadas al Desarrollo Agroforestal, Campus de Rabanales, 14071 Córdoba, Spain; ccima@idaf.es

**Keywords:** imbalanced data, burned area, prediction large wildfire, logistic regression, multi-layer perceptron

## Abstract

Wildfires are becoming more frequent in different parts of the globe, and the ability to predict when and where they will occur is a complex process. Identifying wildfire events with high probability of becoming a large wildfire is an important task for supporting initial attack planning. Different methods, including those that are physics-based, statistical, and based on machine learning (ML) are used in wildfire analysis. Among the whole, those based on machine learning are relatively novel. In addition, because the number of wildfires is much greater than the number of large wildfires, the dataset to be used in a ML model is imbalanced, resulting in overfitting or underfitting the results. In this manuscript, we propose to generate synthetic data from variables of interest together with ML models for the prediction of large wildfires. Specifically, five synthetic data generation methods have been evaluated, and their results are analyzed with four ML methods. The results yield an improvement in the prediction power when synthetic data are used, offering a new method to be taken into account in Decision Support Systems (DSS) when managing wildfires.

## 1. Introduction

Forest ecosystems have an inestimable capacity to sequester carbon dioxide (CO_2_) through time [1], which is very important to the global carbon budget [2]. Droughts, insects and pathogens, landslides, hurricanes, and fires have negative impacts on natural environments [3]. Among these, wildfires are the greatest hazards to forest development [4]. Wildfires can be caused by either anthropogenic or natural causes. Anthropogenic causes, such as carelessness or arson, accumulate the highest percentage of forest fire origins [5,6], negatively impacting economy and quality of life in both local and regional scales, in addition to the harm to natural environments. The average area burned in the world in the last 16 years is about 340 million hectares [7], with the latest report on forest fires in Europe indicating that 178,000 hectares were burned [8]. In addition, the number of countries that have been harmed in some way by large wildfires is higher than ever before. This damage is further exacerbated by the process of climate change that our planet is experiencing. For example, as the intensity and frequency of drought periods increase, the follow-up impact of wildfires increase, both in fire intensity and frequency [9].

In this context, the prediction, prevention, and management actions for wildfires are crucial. Decision Support Systems (DSS) for wildfires are powerful tools to prevent and manage forest fires by providing data for efficient resource use [10]. These DSSs are based mainly in (a) prediction models of geospatial (topography, land uses, infrastructures, among others), satellite and meteorological data [11,12], (b) thematic maps and risk indexes of forest fuel and vegetation [13,14,15], (c) fire propagation and behavior models [16,17,18] and (d) programs for planning and coordination of fire departments (human force, land, and/or aerial machinery) [19,20]. Several wildfire simulators have been developed, which combine algorithms with theoretical forest fire spread. These simulations aid in the prediction of fire spread, which in turn allows for more accurate and effective extinguishing plans. However, wildfire behavior is a complex process involving physical and chemical factors. Multiple models have been developed to explain fire behavior [21,22,23,24,25,26] and multiple simulation algorithms have been developed using Huygens’ principle of wave propagation [27,28], infiltration theory [29], fractal theory [30,31], or cellular automata [32,33,34]. This mathematical modelling of fire is integrated into tools for the simulation of the spread and/or management of wildfires such as FARSITE (Fire Area Simulator-Model Development and Evaluation) [35], Prometheus [17,36], BushFire [37], Fire! [38], FireMaster [39], FireStation [40], or SiroFire [41]. However, there are errors related to the spatial and temporal spread of fire, which are linked to large-scale wildfires [42]. These errors are related to the accuracy of input data and lack of clarity over the relevance of parameters used by algorithms [43].

Wildfire behavior models can be classified with physics-based, statistical, and machine learning (ML) methods. Physics-based methods implement equations of canopy biomass, heat transfer, and fluid mechanics, modeling fire behavior in spatial and temporal dimensions [44,45]. These models demand detailed datasets such as the location and dimensions of trees or fuel mass, which is difficult to register on a large scale [46]. Statistical methods provide adequate models for large areas using different methods such as Poisson regression [47], multiple linear regression [48], logistic regression [49], or Monte Carlo simulation [50] by using data at different scales and resolutions [51]. However, results are not accurate because wildfire spread is a complex and non-linear process. Finally, machine learning methods have been explored, yielding better results, in general, than statistical methods [52]. Among the techniques explored in this group are Random Forest [53], Kernel logistic regression [54], or Artificial Neuronal Networks [55]. Normally, these methods offer better results than those based on statistical models because factors such as temperature, wind, rainfall, slope, orientation or land use interact in multiple and complex forms [56].

While it is necessary to have adequate fire management policies, the majority are focused on fire extinction, with the objective to suppress fire at any cost [57], and they do not address socio-economic and land management issues at the initial phase and spread of wildfire [58]. This strategy depends on budget allocation, and therefore, it has economic limitations. As result, effectiveness to suppress and control a wildfire becomes a problem of the initial attack. It is understood that the best fire-fighting response to control wildfires is restricting their ability to become a large wildfire event. As result, this initial attack is linked to the number and type of resources used at the onset of fire detection [59]. Normally, the initial attack consists of a few fire engines and ground crews, occasionally assisted by aerial vehicles. As a result, fire control is more successful in low wildfire intensity [60]. The number and type of resources used in the initial attack is defined by taking fire risk and fuel moisture indices into account [61,62,63,64]. Initial attack success depends on multiple factors such as weather conditions, early detection, or fire services arrival time [65]. In previous research, initial attack has been addressed using scenario-based models [66], mixed and linear [67], or two-stage stochastic models [68] with the objective of determining optimal fire engine and crew dimensions without taking into account geospatial data [69].

Given this scenario, there is no certainty in the success of initial attack actions. In addition, it should be noted that not all wildfires should be suppressed in this manner [70]. Therefore, it is necessary to identify wildfire events with high probability of becoming large wildfires. Factors driving large wildfires vary in time and space, but it is not clear which factors are best to be used in a predictive model [71]. Previous research has been carried out on this [72,73]; however, there is still uncertainty predicting large wildfires [74]. The objective of this study was to determine a methodology to predict, at the first moment of wildfire detection, if it is going to become a large wildfire using ML techniques to support initial attack planning.

## 2. Materials and Methods

### 2.1. Study Area

**Figure 1:**
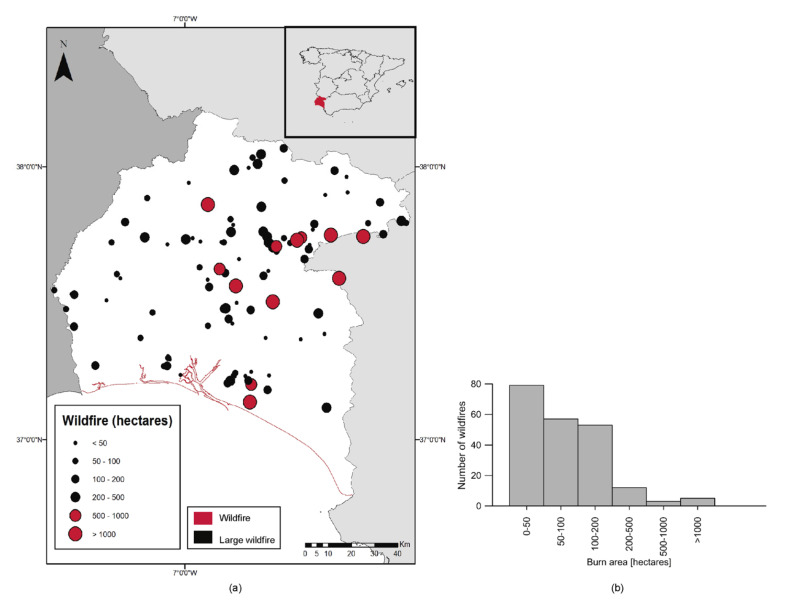
Distribution of wildfires: (**a**) Location of wildfires within study area between 2000 and 2018, and (**b**) histogram of frequency of wildfires by area.

The study area is located in southwestern Spain (37°28′42″ N, 6°54′19″ W, WGS-84), more specifically in western Andalusia, covering the province of Huelva. Every year, there are large recurrent wildfires in the area, defined, in this study, as those that exceed a burn area higher than 500 hectares [75,76]. While in Spain as a whole, the percentage of large wildfires is low, 0.48% in 2017, in Huelva, there is always at least one per year [8]. As an example, in 2018, there were only three large wildfires in Spain, one of which was in the study area [8]. This makes this area of special interest when modeling and predicting the presence of large wildfires. Figure 1a shows the location of 210 wildfires lasting more than 6 h within the study area between 2000 and 2018. The average burnt area surface was equal to 637 hectares, with a maximum equal to 34,290 hectares, which occurred on 27 July 2004. As Figure 1b shows, the number of large wildfires were significantly smaller than normal wildfires. This imbalanced sample makes the results using machine learning techniques biased toward the majority class. For this reason, this study analyzes different sample balancing techniques through the generation of synthetic data.

### 2.2. Data Analysis

A total of 20 variables were analyzed for predicting the occurrence of large wildfires, including meteorological and environmental data as well as data calculated from Landsat and Moderate Resolution Imaging Spectroradiometer (MODIS) scenes [77] (Table 1). This variable selection was based on previous research [78,79,80]. The environmental variables were obtained from the Environmental Information Network of Andalusia (REDIAM) [81]. These variables have an annual–temporal resolution that is far too involved to describe in this paper; however, the details of and process of obtaining these variables can be found at http://www.juntadeandalucia.es/medioambiente/site/rediam (accessed on 17 December 2020). For the meteorological variables, two data sources were used. Firstly, data from the Spanish Meteorological Agency (AEMET) [82] were used to characterize pre-existing variables of the total study area before wildfire incidences, while on-site data provided by the Regional Forest Fire Fighting Plan of Andalusia (INFOCA) [83] were used to define the meteorological conditions of specific wildfire locations. The variables in the study area characterize the seasonal behavior of the year in which wildfires occur. Thus, the following variables were used:

- Burn area mask: binary raster coverage where a value equal to 1 has been assigned to each pixel that has been affected by a wildfire and a value equal to 0 has been assigned to those pixels that have not been affected by a wildfire.
- Normalized Difference Vegetation Index (NDVI) [84]: calculated from the closest Landsat scene for all burn areas, obtained from the Google Earth Engine.
- Land Surface Temperature: obtained from the closest MOD11A1.006 [79], from the MODIS program, for all burn areas, obtained from the Google Earth Engine.
- Fuel model: fuel modeling adapted to the characteristics of the Mediterranean landscape [85].
- Danger index: reflects the probability of wildfire spread once started.
- Fuel model risk index: includes information on forest fuel patterns as well as on those areas that, despite not having forest vegetation, have agricultural crops susceptible to fire spread.
- Watershed’s fuel model risk: represents the mean value of fuel model risk index at watershed scale.
- Geographical risk: measured by slope, annual hours of insolation, and density of drainage points
- Watershed’s surface risk: the mean value of geographical risk at watershed scale.
- Local risk index: refers to the risk of affecting human or heritage elements due to their exposure to wildfire, taking into account urban areas, transport networks, elements of historical heritage, power lines, and fuel pipelines.
- Watershed’s meteorological risk index: shows at watershed scale those factors related to meteorological conditions that will influence the development of the wildfire. These factors are wind speed and vegetation water deficit.
- Water stress risk index: represents humidity state of vegetation based of the relation between rainfall and evapotranspiration.
- Historical risk index: shows the probability of a wildfire occurring as a function of the historical frequency of wildfires.
- Slope risk index: measures the influence on wildfire behavior of slope as it favors the vertical continuity of fuel.
- Forest vulnerability risk: evaluation the quality of forest ecosystems, measuring continuity in terms of total area of tree coverage.
- Wind speed, wind direction, relative humidity, and mean temperature: measured in real time by a weather station site close to a wildfire.
- Water stress: measures humidity content of soil.

**Figure 2:**
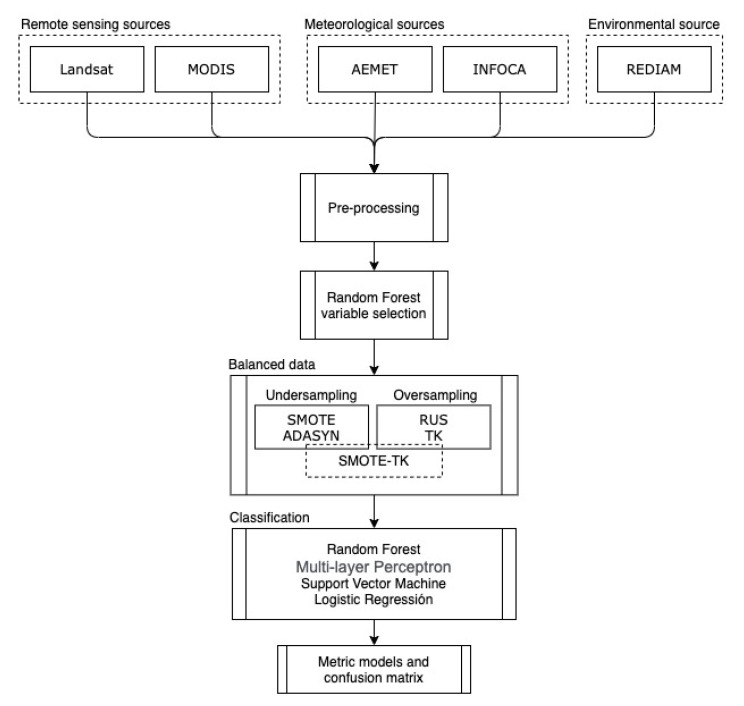
Flowchart used for predicting large fires.

Figure 2 summarizes the workflow for classifying wildfire size. Firstly, data were pre-processed to generate new variables (Risks 1, 2, and 3). From this new dataset, the Random Forest (RF) technique was used for identifying significant variables. Since large wildfires are fewer than those belonging to the general class of wildfires, several techniques to create synthetic datasets were analyzed to balance the sample size of both classes. These new datasets, together with the original data, were used by four types of ML classifiers in order to predict the size of a wildfire. Finally, these results were evaluated to detect which type of synthetic data generation method and prediction model provided the best results. The prediction accuracy of both classes, wildfire and large wildfire, were also analyzed based on omission and commission errors.

The high number of candidate predictor variables and the low number of observations can impact the machine learning results [86], and therefore, the accuracy of the classifier can be overly optimistic, resulting in an overfitted model [87]. To this end, all the risk variables from REDIAM were summarized using three mean risk variables (Risks 1, 2, and 3). First, the variables were grouped according to whether they represented danger (Risk 1), risk associated with the individual watersheds (Risk 2), and risk associated with geography (Risk 3). In the case of Risk 1, only one variable was linked, and the variable was renamed. In the case of the variables within Risks 2 and 3, which were discussed previously using REDIAM, they were calculated by the mean value of the sub-variables for Risks 2 and 3. Then, RF was applied to this new set of variables to determine their individual importance. Variable importance was evaluated through the Gini Index and Out of Bag accuracy, measuring the degree of association between a given variable and the classification result [88].

Since the number of large wildfires (sample size equal to 53) was significantly smaller than the totality of wildfires (sample size equal to 157), data were highly imbalanced with the results being biased toward majority class wildfire. As the classifier model assumes, data are drawn from a balanced distribution, and thus, in this case, they produce undesirable results, which can be resolved by balancing techniques [89] divided into two groups: undersampling and oversampling. The former removes data in the majority class, while the latter generates synthetic data in the minority class to balance the ratio between the two classes. For undersampling methods, Random UnderSampling (RUS) and TomeK link (TK) were used. The RUS algorithm balances the classes through random elimination of instances from the majority class [90], while TK detects pairs of samples of nearest neighbors belonging to different classes [91]. TK can either be used, as in this manuscript, in undersampling (majority samples are removed) or cleaning (both samples are removed) mode [92]. The oversampling methods used were Synthetic Minority Oversampling TEchnique (SMOTE) and ADAptative SYNthetic sampling (ADASYN). With the SMOTE algorithm, the minority class is oversampled by forming convex combinations of neighboring samples [93,94], while ADASYM weighs minority samples according to their level of difficulty of learning [95]. Finally, the Synthetic Minority Oversampling Technique-TomeK link (SMOTE-TK) method was used for balancing. This algorithm applies TK as an undersampling technique on the samples that are generated by SMOTE [96].

Next, four different ML classification algorithms were applied to predict wildfire size: Random Forest (RF) [97], Multi-Layer Perceptron (MLP) [98], Support Vector Machine (SVC) [99], and LOGistic regression (LOG) [100]. A grid search was performed for each classifier to find optimal hyperparameters using Scikit-learn in Python (using GridSearchCV library), as summarized in Table 2. Of the total number of wildfires, 70% were used in the training phase and 30% were used in testing. Training and testing processes have been performed on a virtual machine on Google Colaboratory, which is a free cloud service from Google for machine learning applications, with a Central Processing Unit Intel Xeon 2.30 GHz and a Graphical Processing Unit Tesla K80. Training took less than 9 min and testing only took a few seconds with this configuration.

For the assessment of the ML models, True Positives (TP), True Negatives (TN), False Positives (FP), and False Negatives (FN) were counted in order to calculate accuracy (1), precision (2), recall (3), specificity (4), Geometric-mean (G-mean) (5), and F1-score (6):

- Accuracy: the ratio of correctly predicted observations to the total observations.
  (1) $$\mathrm{Accuracy}=\frac{\mathrm{TP}+\mathrm{TN}}{\mathrm{TP}+\mathrm{TN}+\mathrm{FP}+\mathrm{FN}}$$
- Precision: the ratio of correctly predicted positive observations to the total predicted positive observations.
  (2) $$\mathrm{Precision}=\frac{\mathrm{TP}}{\mathrm{TP}+\mathrm{FP}}$$
- Recall: measures how well the classifier can detect positive observations.
  (3) $$\mathrm{Recall}=\frac{\mathrm{TP}}{\mathrm{TP}+\mathrm{FN}}$$
- Specificity: measures how well the classifier can detect negative observations.
  (4) $$\mathrm{Specificity}=\frac{\mathrm{TN}}{\mathrm{TN}+\mathrm{FP}}$$
- G-mean: equal to the geometric mean of recall and specificity, this shows the balance between classification on the majority and minority class.
  (5) $$G-\mathrm{mean}=\sqrt{\mathrm{Recall}\cdot \mathrm{Specificity}}$$
- F1-score: the harmonic average of precision and recall.

In addition, omission and commission errors were calculated to analyze the results per wildfire class.

(6) $$F1-\mathrm{score}=2\cdot \frac{\mathrm{precision}\cdot \mathrm{recall}}{\mathrm{precision}+\mathrm{recall}}$$

## 3. Results

**Figure 3:**
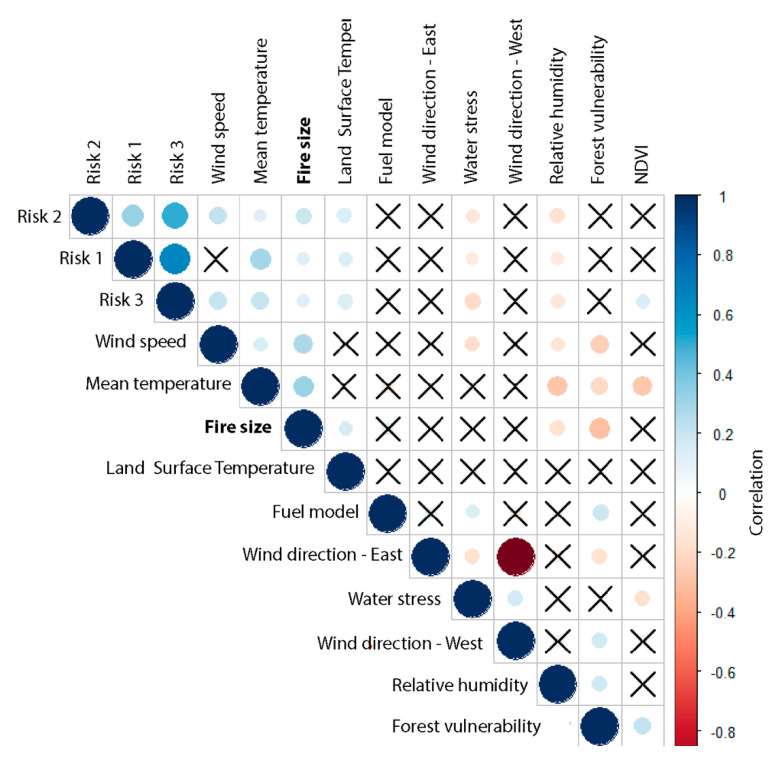
Correlation matrix of variables (significance level for Pearson lower than 0.05; X not significant).

As in the previous analysis, the correlation coefficient between all paired variables used in this study is shown in Figure 3, where positive correlation is represented in blue and negative correlation is represented in red. In addition, color intensity and the circle size are proportional to the correlation coefficients. Wildfire size showed significant correlation with wind speed, LST, mean temperature, risks 1, 2, and 3, forest vulnerability, and relative humidity. In addition, several fuel models were created for wildfire prediction, taking into account plant characteristics and their influence on speed and intensity of flame propagation, as proposed by Rothermel [101]. However, the fuel model variable did not indicate any relationship with wildfire size or the other variables. This explains the lack of classification or mapping of the fuel model in this study. Similar results have been found in other research projects in Spain [102].

**Figure 4:**
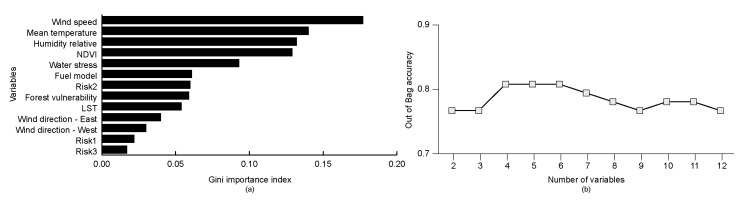
Selection of variables based on (**a**) Gini Index and (**b**) Out of Bag accuracy results.

The Gini importance index results with the potential predictor variables are shown in Figure 4a. Wind speed and mean temperature were the most important variables, while risk 1 and risk 3 were the least important. On the other hand, Figure 4b shows that Out of Bag accuracy performs best with four variables. Based on these results, wind speed, mean temperature, relative humidity, and NDVI were the four selected predictor variables in this study.

Once the variables were selected, different synthetic data generation methods were applied to balance the sample. Table 3 shows the sample size of each dataset generated.

**Figure 5:**
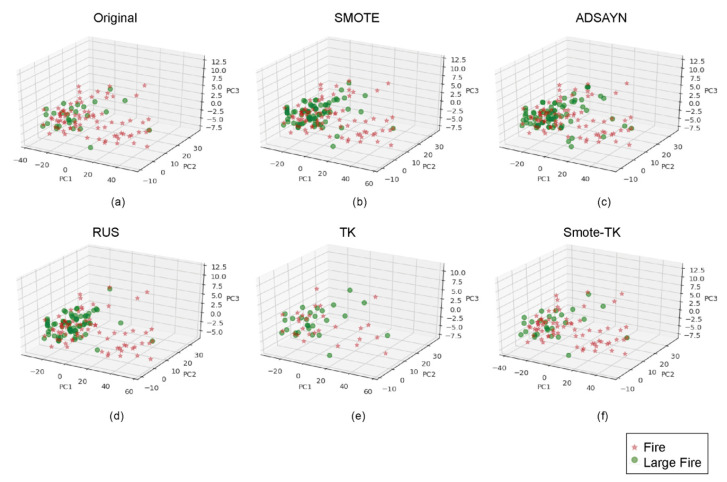
Three-dimensional PCA results per original and synthetic datasets: (**a**) Original data, (**b**) SMOTE, (**c**) ADASYN, (**d**) RUS, (**e**) TK and (**f**) Smote-TK.

**Figure 6:**
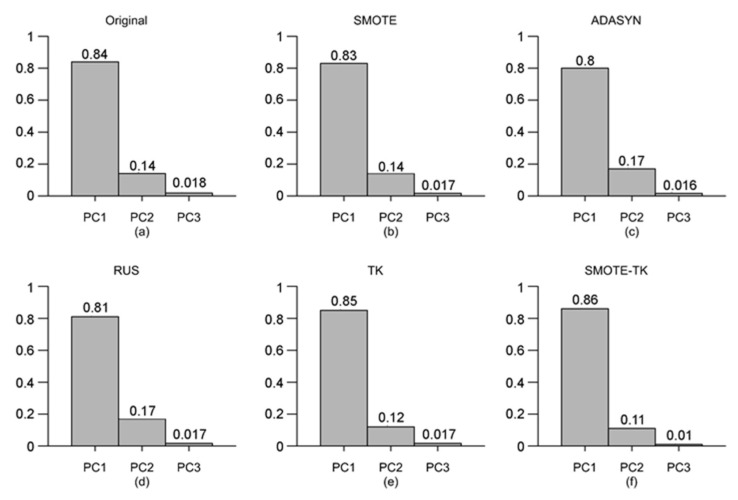
Principal components ranked by variance per original and synthetic datasets: (**a**) Original data, (**b**) SMOTE, (**c**) ADASYN, (**d**) RUS, (**e**) TK and (**f**) Smote-TK.

Figure 5 shows a three-dimensional training sample of wildfires (red star) and large wildfires (green circle) throughout a PCA analysis of selected variables for each undersampling and oversampling methods (Figure 5b,f) applied and original data (Figure 5a). On the other hand, Figure 6 shows principal components per dataset. For the undersampling methods, PCA-SMOTE (Figure 6b) generated homogenous distributed synthetic data around the large wildfire region. This region increased with PCA-ADSAYN (Figure 6c). For the oversampling methods, PCA-RUS (Figure 6d) removed data from the wildfire majority class, showing greater differentiation between both classes compared to TK-PCA (Figure 6e). Finally, in Smote-TK-PCA (Figure 6f), wildfires and large wildfires were well differentiated but not as well as in PCA-SMOTE and ADASYN.

**Figure 7:**
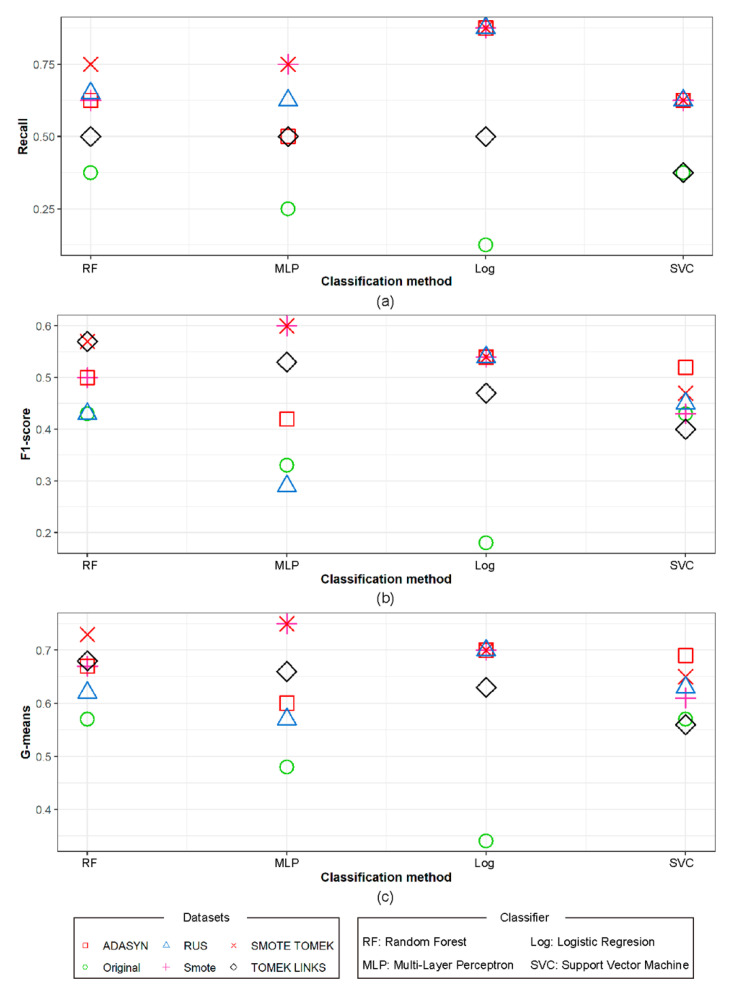
Accuracy of wildfire size prediction based on (**a**) Recall, (**b**) F1-score, and (**c**) G-means.

Alongside the original dataset, the resulting quality of wildfire size prediction using RF, MLP, Log, and SVC models throughout Recall, F1-score, and G-means are shown in Table 4 and Figure 7. Based on the Recall parameter, the Log model showed the best results. In addition, RUS, SMOTE TOMEK, SMOTE, and ADASYN were the best methods for generating synthetic data for this model, while TOMEK LINKS showed the worst result, yielding similar recall values to the original data using SVC. On the other hand, MLP using SMOTE and SMOTE TOMEK data yielded the best results in the F1-score. As before, the original data gave the worst results. The same results appear with the G-means parameter. The original unbalanced data alone did not provide better results than those described above, therefore showing the advantage of using synthetic data in order to improve wildfire size prediction.

**Figure 8:**
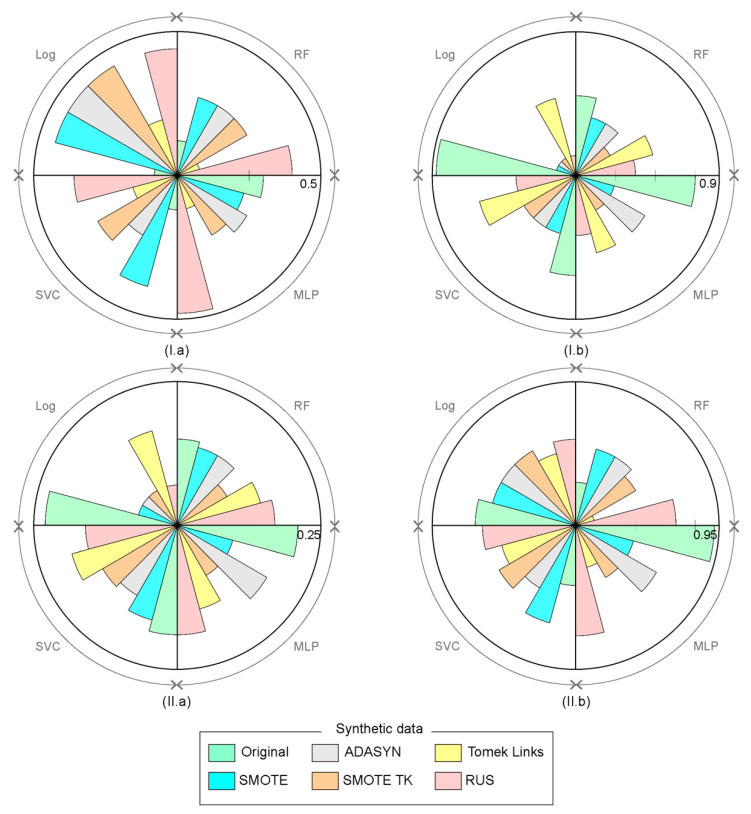
Omission (**I**) and commission (**II**) error for wildfire (**a**) and large wildfire (**b**) classes.

The overall prediction results above were discussed without considering the errors obtained in the analysis of the two wildfire classes. Figure 8 shows the errors of omission and commission for wildfire and large wildfire classes for each model and dataset used. In general, omission errors in the prediction of wildfires (Figure 8(I.a)) were greater than those for large wildfires (Figure 8(I.b)), while on the other hand, the commission error was lower for large wildfires (Figure 8(II.b)) than wildfires overall (Figure 8(II.a)). The lowest omission error in predicting wildfires (Figure 8(I.a)) was obtained when the original data were used regardless of the model applied. However, if synthetic data were used in the same case, the number of wildfires predicted as large wildfire increased. Contrariwise, the omission error in large wildfire prediction decreased if synthetic data were used (Figure 8(I.b)). In this case, the Log model using SMOTE, ADASYN, SMOTE TK, and RUS gave the best results and therefore offered a greater accuracy in predicting large wildfires. On the other hand, the commission error for wildfires (Figure 8(II.a)) was low in those cases where the Log model was applied, using SMOTE, ADASYN, SMOTE TK and RUS synthetic methods. Here, using original data showed the worst results. Finally, MLP using SMOTE and SMOTE TK and RF using TOMEK LINK and original data gave a low commission error (Figure 8(II.b)).

## 4. Discussion

ML methods applied in studies of burn-area prediction are relatively novel compared to other wildfire applications. To date, these methods are applied to forecast or predict the total area burned and fire occurrence [103,104]. However, these results do not take into account the environmental conditions at the time a wildfire starts. In the proposed methodology, a total of 20 variables have been evaluated to predict the occurrence of a large wildfire. Of these, four have been selected: wind speed, mean temperature, relative humidity, and NDVI. Each of these are linked to real-time or near real-time data. The first three variables coming from a weather station installed close to the wildfire location and the last variable from the most recent Landsat scene at the time of the wildfire, allowing the prediction to be adapted to what is happening at that precise moment of the fire. The selection of meteorological variables is similar to those selected in previous research [74].

The fuel model variable was not selected, although it has been used in previous research projects related to wildfires [105,106]. Our results on fuel models were not conclusive, which was likely due to the low temporal resolution of these data—an aspect that has been detected by other authors working in the same region [102].

Previous studies to model burn area mainly use multiple linear regression models [48,107]; however, there are not many studies using ML techniques in this field [55,108,109]. These studies make predictions of wildfire probability without taking into account the environmental conditions at the onset of the fire. On the other hand, many large wildfire predictions are suboptimal, as they are concentrated in small regions where no general models fit appropriately, which is mostly likely due to small numbers of large wildfires [110]. Therefore, the prediction of large wildfires presents difficulties as they are uncommon events with respect to overall wildfire occurrence. Earlier research, as [111], propose the need to establish a threshold to delimit a large fire event [112]; however, this threshold is debatable [113]. On the other hand, recent ML-based models at continental or global scales for predicting burn areas offer good results in general term but fail to distinguish large wildfires [114]. This imbalance of data justifies the use of synthetic data as proposed in this project.

Based on our results and considering the effectiveness and efficiency, Log and MLP were the best-performing models. In the case of the Log model, it yielded very low errors of omission in the prediction of large wildfires when synthetic data were used, except when using Tomek Links. However, this model was not the most effective, as the wildfire omission error resulted in the highest value of errors. Thus, the model adopts a conservative profile so that the necessary resources will always be mobilized for a large wildfire, assuming that on some occasions, the resources will be oversized. On the other hand, using MLP as a predictive model and SMOTE and SMOTE TK as a technique to generate synthetic data will make the response more efficient but slightly less effective. Thus, the error of omission for a large wildfire is slightly increased, but the error in wildfires will be smaller. Finally, the use of the original data is not recommended, mainly because of the high number of omissions in the prediction of a large fire, indicating the need to balance the sample.

In this study, the use of machine learning applications based on synthetic data to generate a predictive model of the presence of a large wildfire in the early stages has been evaluated. Of all the variables analyzed, the most important were those with a very high temporal resolution rather than historical variables, and therefore, the deployment of sensors over the wildfire area is highly recommended in the initial phase of extinction in order to monitor temperature and wind speed. On the other hand, although the fuel model variable has not been selected in this study, future work should use updated fuel model data to improve the results. Furthermore, we propose the evaluation of this methodology on a large working area, at country or continent scale, to assess its suitability.

## 5. Conclusions

Wildfires are one of the most dangerous natural hazards across the world and, for this reason, any effort to support its analysis and management is important. Knowing at an early stage whether a wildfire is going to become a large wildfire permits better management of human and material resources. In this study, the use of machine learning methods together with the appropriate selection of variables have provided satisfactory results in the prediction of large wildfires. For this, the selection and processing of data is one of the most important aspects. In this context, the analysis carried out has shown that those data registered at the time of the wildfire were more important than those based on historical series and that it is necessary to balance the data sample due to the higher occurrence of wildfires compared to large wildfires. Given the promising results presented here, the proposed methodology will be applied in future campaigns and will be extended to other regions.

## Figures and Tables

**Table 1:** Variables analyzed in the prediction of the occurrence of a large fire.

Variables	Sub-Variable	Type	Source	Temporal Resolution
Burn area mask		Binary categorical	INFOCA	Annual
NDVI		Numerical	Landsat	Near real time
Land Surface Temperature		Numerical	Modis	Near real time
Fuel model		Categorical	REDIAM	Annual
Danger index	Risk 1	Numerical	REDIAM	Annual
Watershed’s fuel model risk	Risk 2	Categorical	REDIAM	Annual
Watershed’s surface risk		Categorical	REDIAM	Annual
Local risk index		Categorical	REDIAM	Annual
Wathersed’s meteorological risk index		Categorical	REDIAM	Annual
Fuel model risk index		Categorical	REDIAM	Annual
Water stress risk index		Categorical	REDIAM	Annual
Historical risk index		Categorical	REDIAM	Annual
Slope risk index	Risk 3	Categorical	REDIAM	Annual
Geographical risk		Cateogorical	REDIAM	Annual
Forest vulnerability risk		Numerical	REDIAM	Annual
Wind speed		Numerical	INFOCA	Real-time
Wind direction		Categorical	INFOCA	Real-time
Water stress		Numerical	AEMET	Near real-time
Relative humidity		Continuous	INFOCA	Real-time
Mean temperature		Continuous	INFOCA	Real-time

**Table 2:** Optimized hyperparameters per classifier and dataset.

Classifier	Hyperparameter	Original	SMOTE	ADASYN	SMOTE TK	TK	RUS
Random Forest	Criterion	Gini	Gini	Entropy	Entropy	Entropy	Gini
	Maximum number of features	4	7	6	7	4	4
	Maximum of level in tree	Automatic	SQRT	Log2	Log2	SQRT	SQRT
	Number of tress	200	200	200	500	500	200
Multi-layer Perceptron	Activation	Identity	Logistic	Logistic	Logistic	Identity	Identity
	Alpha	0.005	0.005	0.01	0.005	0.0001	0.0001
	Hidden layer sizes	50	50	50	50	100	10
	Learning rate	Adaptative	Constant	Constant	Adaptative	Constant	Constant
	Solver	SGD	LBFGS	LBFGS	LBFGS	SGD	SGD
Support Vector Machine	Regularization parameter	100	100	1000	10	100	10
	Gamma	0.0001	0.0001	0.001	0.001	0.0001	0.0001
	Coefficient kernel	RBF	RBF	RBF	RBF	RBF	RBF
Logistic Regression	Inverse of regularization parameter	1	1	1	1	1	1
	Penalty	l2	l2	l2	l2	l2	l2

**Table 3:** Sample size of wildfire and large wildfire per original and synthetic datasets.

Dataset	Wildfire	Large Wildfire
Original	189	21
SMOTE	189	189
ADASYN	189	189
RUS	52	52
TK	134	48
SMOTE-TK	171	172

**Table 4:** Accuracy and 95% confidence interval of wildfire size prediction based on (a) Recall, (b) F1-score, and (c) G-means.

Accuracy Parameter	Classifier	Random Forest	Multi-Layer Preceptron	Support Vector Machine	Logistic Regression
Recall	Original	0.37 (0.32–0.75)	0.25 (0–0.70)	0.37 (0–0.61)	0.12 (0.04–0.60)
	SMOTE	0.62 (0.61–0.96)	0.75 (0.63–0.95)	0.62 (0.41–0.91)	0.87 (0.49–0.91)
	ADASYN	0.62 (0.61–0.97)	0.50 (0.45–0.95)	0.62 (0.56–0.96)	0.87 (0.43–0.92)
	SMOTE TOMEK	0.70 (0.72–0.99)	0.75 (0.66–0.95)	0.62 (0.48–0.93)	0.87 (0.52–0.90)
	TOMEK LINKS	0.5 (0.41–0.90)	0.52 (0.11–0.88)	0.37 (0.09–0.69)	0.51 (0.14–0.71)
	RUS	0.65 (0.49–1)	0.62 (0.42–0.97)	0.62 (0.15–0.98)	0.87 (0.32–0.97)
F1-Score	Original	0.42 (0.36–0.75)	0.33 (0.05–0.63)	0.42 (0–0.62)	0.18 (0.07–0.62)
	SMOTE	0.5 (0.45–0.75)	0.6 (0.58–0.88)	0.43 (0.42–0.76)	0.53 (0.51–0.75)
	ADASYN	0.5 (0.46–0.91)	0.42 (0.41–0.89)	0.52 (0.48–0.88)	0.53 (0.52–0.77)
	SMOTE TOMEK	0.57 (0.45–0.92)	0.6 (0.42–0.90)	0.47 (0.45–0.79)	0.53 (0.50–0.79)
	TOMEK LINKS	0.57 (0.48–0.82)	0.53 (0.22–0.77)	0.4 (0.16–0.75)	0.47 (0.23–0.74)
	RUS	0.43 (0.43–0.86)	0.29 (0.29–0.84)	0.45 (0.25–0.77)	0.53 (0.38–0.77)
G-means	Original	0.57 (0.38–0.80)	0.48 (0.10–0.72)	0.57 (0–0.71)	0.33 (0–0.58)
	SMOTE	0.67 (0.48–0.85)	0.75 (0.57–0.87)	0.61 (0.43–0.85)	0.75 (0.5–0.90)
	ADASYN	0.67 (0.55–0.92)	0.6 (0.49–0.89)	0.68 (0.51–0.92)	0.73 (0.51–0.82)
	SMOTE TOMEK	0.73 (0.61–0.95)	0.75 (0.59–0.93)	0.65 (0.48–0.82)	0.76 (0.5–0.79)
	TOMEK LINKS	0.67 (0.41–0.81)	0.66 (0.19–0.90)	0.56 (0.21–0.72)	0.63 (0.17–0.70)
	RUS	0.62 (0.45–0.98)	0.57 (0.35–0.91)	0.63 (0.20–0.85)	0.72 (0.34–0.87)

## Data Availability

Data generated in this study are available from the corresponding author.
